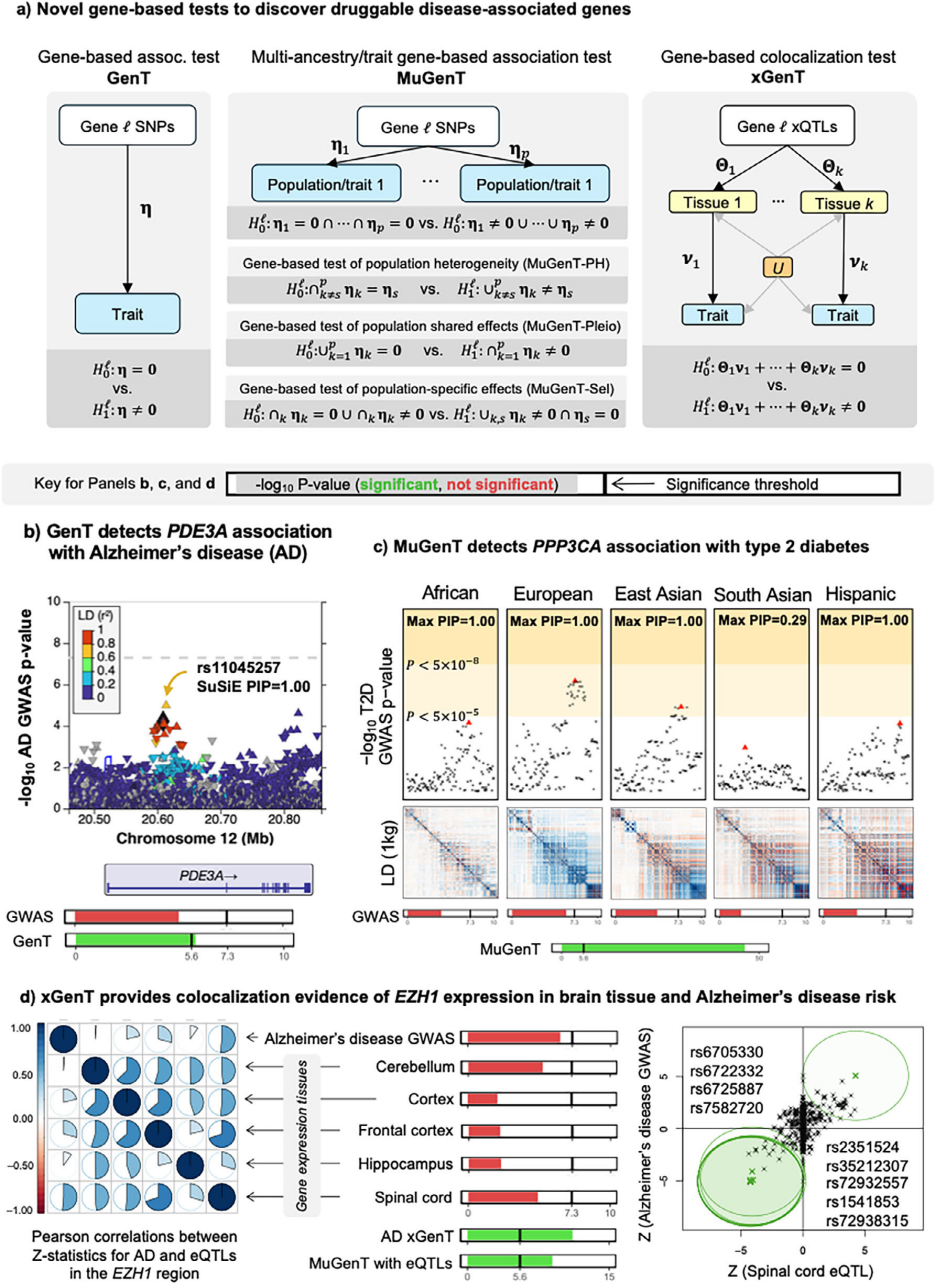# Estimating shared polygenicity identifies novel druggable genes for Alzheimer's disease

**DOI:** 10.1002/alz70855_102953

**Published:** 2026-01-05

**Authors:** Noah J Lorincz‐Comi, Feixiong Cheng

**Affiliations:** ^1^ Cleveland Clinic, Cleveland, OH, USA; ^2^ Cleveland Clinic Genome Center, Cleveland, OH, USA

## Abstract

**Background:**

Identifying genes which are associated only with Alzheimer's disease (‘AD exclusive’) vs those which are additionally associated with other phenotypes (‘AD pleiotropic’) has been historically challenging because of a lack of inferential approaches. AD exclusive genes may be potential drug targets because of their lower potential for off‐target side effects, and AD pleiotropic genes may be repurposable drug candidates if the directions of association are in protective directions for the other traits and AD.

**Method:**

We introduce a general Bayesian method which returns posterior probabilities (PP) that a gene is AD exclusive or AD pleiotropic by estimating its shared polygenic architecture with multiple other phenotypes. This method requires only gene‐based association test statistics from summary level genome‐wide association study (GWAS) data. We searched for AD exclusive and pleotropic genes among a set of 16,232 and considered 31 other complex disease and cardiometabolic phenotypes with which AD may associate.

**Result:**

We estimate that at least 367 genes independently associate with AD, of which only 17 (4.6%) can be targeted with existing drugs and are inferred to be AD exclusive (PP>0.5; e.g., *INPP5D, CD55, TAS2R41*). For example, the posterior probability that *INPP5D* was associated with AD was 0.81 and with *any* of the 31 other traits was only 0.23. Alternatively, 140 (38.1%) druggable genes are estimated to belong in the AD pleiotropic set (PP>0.5; e.g., *CLU, APOA2, APOC2, LIPC, APOE*). For example, *APOA2* had posterior probability of likely causing AD of 0.97 and for Bipolar I/II (BPD) of 0.83. In this locus, the genetic correlation between AD and BPD was 0.22 (*p* <2E‐16), suggesting that targeting of *APOA2*, which is a member of the IL‐12 pathway that is differentially expressed in BPD patients receiving lithium treatment, may be a repurposable candidate for AD prevention.

**Conclusion:**

The set of AD exclusive genes which are viable druggable candidates is likely very small. This highlights the utility of evaluating the set of repurposable candidates, which we show may be significantly larger. Together, our work highlights the power of our method to identify putatively safer and efficacious drug targets for AD and other AD‐related dementia if broadly applied.